# Field cancerization in urothelial carcinoma: molecular alterations and clinical implications

**DOI:** 10.7150/jca.125266

**Published:** 2026-08-10

**Authors:** Xinni Ye, Junhao Chen, Hongqiong Li, Haonan Dong, Tianjiao Luo, Peng Chen, Haifeng Wang, Shi Fu

**Affiliations:** Department of Urology, The Second Affiliated Hospital of Kunming Medical University, Yunnan, 650106, China.

**Keywords:** Urothelial carcinoma, Field cancerization, Peritumoral tissue, Microenvironment, Gene mutations

## Abstract

Urothelial carcinoma (UC) is a common malignancy of the urinary tract and is characterized by frequent recurrence, particularly in patients with non-muscle-invasive disease. Recurrence has traditionally been explained by residual tumor cells, intraluminal seeding, or the outgrowth of disseminated clones. However, molecular studies have shown that urothelium that appears normal under routine histological examination may already harbor cancer-related alterations. These findings support the concept of field cancerization, whereby prolonged exposure to carcinogens, chronic inflammation, and other injurious stimuli produces a biologically altered urothelial field before overt malignancy becomes visible.

In this review, we searched PubMed and Web of Science for relevant studies published up to January 2026. We focused on investigations of peritumoral tissue or histologically normal-appearing urothelium that reported molecular abnormalities associated with UC development or recurrence. Available evidence from sequencing, methylation profiling, spatial mapping, and multi-omics studies indicates that field changes may involve somatic mutations, copy-number alterations, epigenetic dysregulation, metabolic reprogramming, and remodeling of the local immune and stromal microenvironment. These changes may create a permissive background for clonal expansion, multifocal tumor development, and recurrence after treatment.

Although the clinical definition and spatial extent of the urothelial field remain unsettled, molecular characterization of apparently uninvolved urothelium may help refine risk assessment beyond conventional histopathology. In particular, perioperative urinary tumor DNA testing may assist in identifying molecular residual disease and support more individualized surveillance or adjuvant treatment decisions.

## 1. Introduction

Urothelial carcinoma (UC) arises from the urothelial lining of the renal pelvis, ureter, bladder, and urethra. Among these sites, bladder cancer (BC) accounts for more than 90% of UC cases, and approximately 75% of patients present with non-muscle-invasive bladder cancer (NMIBC) at diagnosis 1. Although cystoscopy, urine cytology, and molecular testing have improved clinical management, recurrence remains common, especially in NMIBC. A proportion of patients also experience disease progression, which creates a substantial long-term treatment and surveillance burden 2, 3.

Traditionally, local recurrence has been explained by persistent tumor cells that remain after treatment or by implantation of exfoliated tumor cells within the urothelial tract. This interpretation is supported by studies showing shared genomic alterations among lesions detected at different times or locations, suggesting that they originated from a common ancestral clone 4. On this basis, clinical assessment and risk stratification still rely largely on pathological findings, including recognizable precursor lesions such as dysplasia and carcinoma in situ 5-7. However, histopathology alone cannot fully explain several clinical observations. Molecular abnormalities have been detected in urothelium that appears normal under the microscope, and some patients develop multifocal tumors at anatomically distinct sites. In addition, urothelial areas considered uninvolved at the time of surgery may later undergo malignant transformation 8-12. These findings have drawn attention to field cancerization (FC), also described as the field effect or field defect.

**Figure 1:**
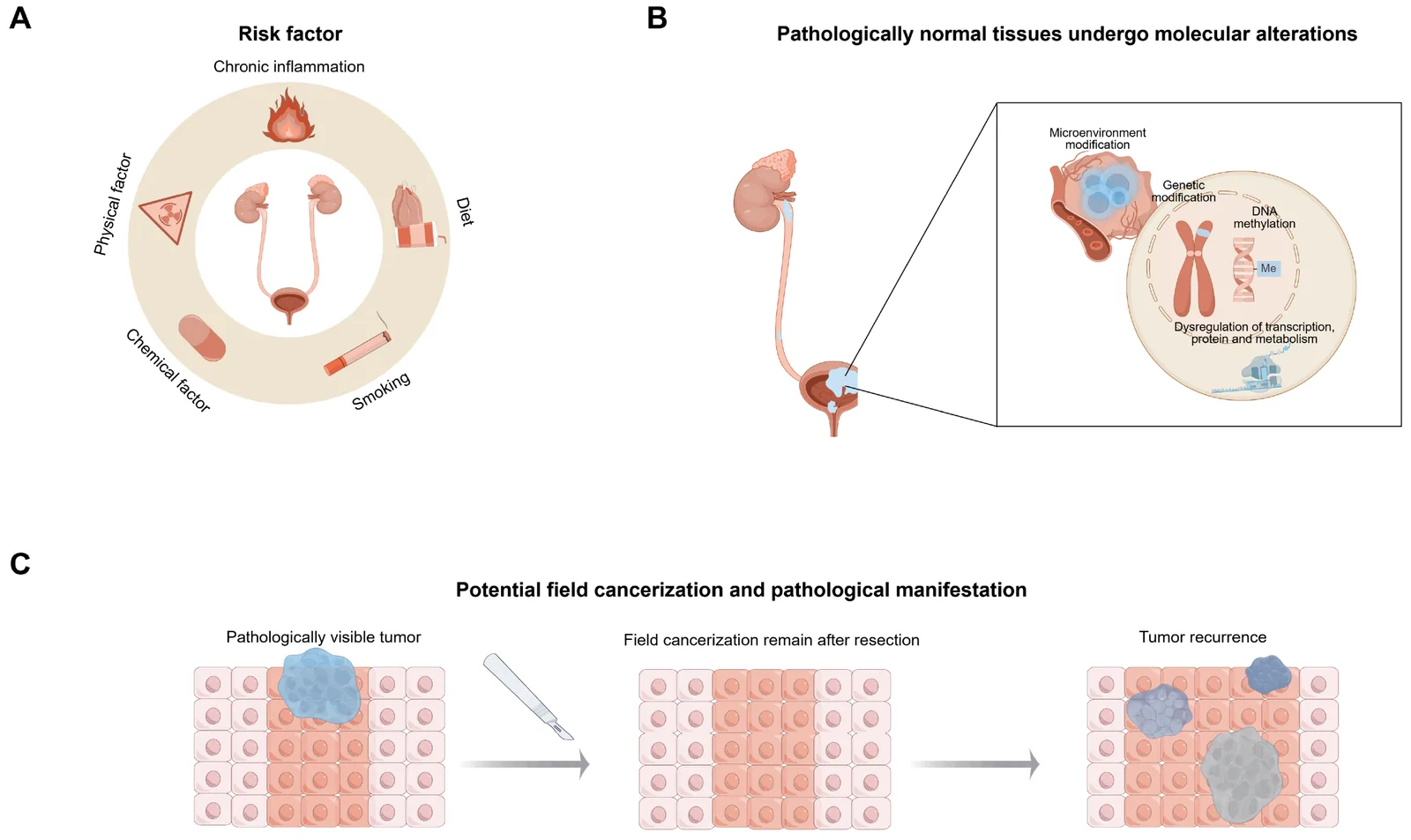
Schematic illustration of the initiation and progression of urothelial carcinoma. (A) Chronic inflammation, unhealthy diet, smoking, as well as physical and chemical carcinogens, are major risk factors for urothelial carcinoma. (B) Under the influence of these carcinogenic factors, histologically normal urothelial epithelium may develop a pathologically invisible field effect, characterized by molecular abnormalities such as genetic alterations, DNA methylation, dysregulation of transcription, translation, and metabolic regulation, accompanied by modifications in the tumor microenvironment. (C) As the disease progresses, urothelial tissues may develop pathologically detectable tumors. Even after tumor resection, the field effect persists, and residual abnormal cells may undergo further malignant transformation, potentially leading to tumor occurrence. By Figdraw.

FC refers to molecular alterations that accumulate in tissue after prolonged exposure to carcinogens, inflammation, or other chronic insults, before visible morphological abnormalities develop 13. In the urothelium, such changes may create a biologically altered background that is more susceptible to malignant transformation. Some regions within this altered field may subsequently acquire additional abnormalities and progress toward dysplasia, carcinoma in situ, invasive disease, or clinically detectable recurrence (Figure 1B) 14-16.

Several established UC risk factors may contribute to this process, including aromatic amines associated with smoking and occupational exposure, arsenic-contaminated drinking water, and recurrent urinary tract infections (Figure 1A) 3. Long-term exposure to these factors may help explain the multifocal nature and high recurrence rate of UC. Notably, some recurrent lesions show molecular patterns that cannot be readily accounted for by simple clonal spread, providing support for the field cancerization hypothesis 17, 18. The bladder is particularly suitable for studying this phenomenon because it is the most commonly affected site and allows sampling of adjacent and distant areas of urothelium within the same organ 19.

This review examines the potential contribution of field cancerization to UC initiation, progression, and recurrence. Relevant studies were identified through PubMed and Web of Science searches conducted up to January 2026 using the terms “field cancerization” and “urothelial carcinoma,” followed by manual screening of relevant records.

## 2. Overview of field cancerization

In 1953, Slaughter and colleagues introduced the concept of field cancerization while studying oral squamous cell carcinoma. They observed that recurrence could not always be explained by the regrowth of the primary tumor alone. Instead, adjacent epithelium often showed gradual proliferative changes and atypical cellular features, suggesting that a broader area of tissue had already been altered before a new lesion became clinically apparent 20. Under this model, removal of the visible tumor does not necessarily eliminate all biologically abnormal cells. Cells remaining in the surrounding mucosa may continue to accumulate changes and, in some cases, give rise to additional tumor foci or later recurrence (Figure 1C) 21.

The meaning of a “cancerized field” is less clearly standardized in urothelial carcinoma. In most studies, it is inferred when histologically normal-appearing urothelium contains molecular features associated with cancer. Consequently, the boundaries of the field are defined differently across studies. Some investigators compare tissue close to the tumor with samples collected from more distant urothelial sites, asking whether tumor-related alterations extend beyond the grossly visible lesion 22. Other studies use whole-organ or multi-region sampling to map molecular changes across anatomically documented areas of the bladder mucosa. More recently, spatial profiling and spatial multi-omics approaches have added positional information to these analyses, allowing molecular features to be interpreted in relation to their precise location within the urothelium 16, 23. Differences in sampling distance, mapping granularity, and analytical platforms therefore introduce inherent challenges when comparing the extent and intensity of field effects across studies.

It is noteworthy that the field cancerization theory does not contradict the traditional theory of clonal expansion. In situ or early recurrence is generally considered to result from the rapid proliferation of the residual tumor clone at the primary site, which is a direct consequence of incomplete surgical and therapeutic eradication, molecularly manifested as monoclonal expansion. In contrast, distant or late recurrence may be promoted by extensive accumulation of field effect, accompanied by subclonal expansion, cooperation among multiple clones, and tumor metastasis 4, 24.

## 3. Molecular alterations of field cancerization

### 3.1 Genetic abnormalities

Field cancerization implies that somatic alterations can be detected in histologically normal urothelium. Evidence for such alterations comes from multiple experimental designs with different strengths. Bulk / targeted sequencing of normal biopsies has shown that somatic variants are common even when urothelium appears normal under microscopy. For example, in a targeted panel study of NMIBC, 67% normal urothelium specimens harbored ≥1 somatic mutation, and TERT promoter mutations were detected in 61% of normal samples 25. A complementary design—deep-targeted sequencing with error correction in selected-site biopsies (SSBs) quantified field effects across multiple bladder locations and time points, and reported that 33% of high-impact mutations in cancer driver genes observed in tumor samples were already present in normal SSBs 15. Spatial/whole-organ multi-omics mapping provides anatomically resolved evidence that molecular changes can extend beyond visible lesions. Whole-organ histologic and genomic mapping combined bulk RNA-seq, methylation profiling, copy-number variation, and exome mutational profiles to track progression from field effects to carcinoma along luminal and basal trajectories 16. Copy number and allele-specific analyses (including CN-LOH) capture alterations that point-mutation panels may miss. Using a multi-sample-per-patient protocol and a sensitive pipeline, one cohort reported that 45% of patients had at least one chromosomal alteration in at least one “non-cancerous” urothelium sample, with recurrent hotspots comprising gains/deletions and CN-LOH events 22.

Across studies, recurrent alterations in histologically normal urothelium tend to involve early trunk-like events inferred by shared mutations between normal and tumor, or chromosomal hotspots recurrently observed across patients. In NMIBC targeted sequencing, TERT promoter and FGFR3 alterations were repeatedly observed in normal urothelium, supporting their role as early events compatible with clonal expansion in NMIBC 25. In the SSB deep-targeted design, 33% of tumor-derived high-impact driver mutations were detectable in normal SSBs, including tumour protein p53 (TP53), AT-rich interactive domain-containing protein 1A, lysine methyltransferase 2D, and phosphatidylinositol-4,5-bisphosphate 3-kinase catalytic subunit alpha 15. Current studies on the mechanisms of field cancerization indicate that most involved molecules are associated with traditional oncogenes or tumor suppressor genes, but some atypical genes and pathways may also be involved. To explore the key alleles affecting tumor development, Wiktoria Stańkowska et al. analyzed the copy-neutral loss of heterozygosity pattern in the normal urothelium of BC patients, combined with chimeric chromosome amplifications and deletions, and identified several altered gene hotspots. These include genes involved in cell cycle regulation, receptor tyrosine kinases that promote the development of a variety of cancers, and several genes participating in the phosphatidylinositol 3-kinase/protein kinase B, mitogen-activated protein kinase, and wingless/integrase-1 signaling pathways. Many of these genes align with BC, for example, the amplification of E2F transcription factor 3, SRY-related high-mobility-group box 4, fibroblast growth factor receptor 1, and zinc finger protein 703 may be associated with the activation of oncogenes, while deletions of SWI/SNF-related matrix-associated actin-dependent regulator of chromatin subfamily A member 2, nuclear factor I/B, and protein tyrosine phosphatase receptor delta may impair tumor suppression functions. Furthermore, the study also observed some rare genetic variations in BC, such as duplication of fibroblast growth factor receptor 1 and deletion of cyclin-dependent kinase inhibitor 1B, providing new research clues regarding urothelial carcinoma pathogenesis 22. Notably, the frequency and types of gene mutations in field cancerization show significant heterogeneity across different studies. Some studies suggest that, compared to classical oncogenes, mutations in genes related to chromatin remodeling are more frequent in field cancerization 26, 27. This phenomenon may be related to the stability of chromatin remodeling genes in tumor development, while mutations in classical oncogenes such as TP53 exhibit more pronounced temporal variation 28.

Importantly, functional evidence in adjacent normal urothelium is usually indirect, most studies do not perform perturbation experiments in normal tissue. Instead, they infer the biological relevance of these alterations by correlating known tumor-suppressor or oncogene functions in bladder cancer with their presence in both normal and tumor compartments. Shared alterations between adjacent normal urothelium and tumor tissue are consistent with clonal continuity and may indicate that some mutations occur early during tumor development. Their relationship with clinical features, including recurrence, progression, or prognosis, may provide further indirect support for biological relevance. Nevertheless, most evidence from adjacent normal tissue remains observational. For example, TP53 inactivation is strongly linked to aggressive bladder cancer, but studies of morphologically normal urothelium generally demonstrate mutation presence or clonal relatedness rather than direct functional effects in experimental models 15, 29.

Molecular changes associated with field cancerization are not uniform across patients, anatomical sites, or molecular subtypes. Different altered urothelial regions may follow distinct evolutionary trajectories, which may contribute to both inter-patient variation and heterogeneity among tumors arising within the same patient 25, 27, 30-33. Prior radiation exposure may also influence the mutational landscape in a subset of bladder cancers. In radiation-associated cases, alterations in KDM6A and ATM, together with PPARG amplification, have been reported more frequently, whereas CDKN2A mutations appear more common in tumors without a history of radiation exposure 34. These findings indicate an association between radiation history and particular genomic patterns, but they do not establish that radiation directly produced the observed mutations or the more aggressive clinicopathological features reported in some cohorts.

Mutation frequencies and reported gene lists also differ considerably between studies. Such variation may reflect differences in patient selection, disease stage, tissue sampling, sequencing depth, and analytical strategy. In particular, some deep-targeted sequencing studies examined only mutations already identified in the matched tumor. This approach can increase the apparent proportion of shared mutations while making it harder to detect additional field-associated alterations that were not present in the index tumor 15.

### 3.2 Epigenetic changes

Field cancerization is not solely driven by genetic mutations; epigenetic regulation also plays an important role. This includes DNA methylation, histone modifications, and chromatin remodeling, among other mechanisms 35, 36. In urothelial carcinoma, DNA methylation has received particular attention. One practical reason is that methylation changes can be detected not only in tissue samples but also in urine-derived DNA, which makes it possible to explore non-invasive biomarkers and to follow molecular changes over time in a longitudinal manner 37, 38. DNA methylation primarily occurs in CpG islands within gene promoter regions and is a chemical modification involving the addition of a methyl group to the 5' carbon of cytosine residues. By changing the structure of DNA, methylation can inhibit gene transcription 39.

In samples from patients with urothelial carcinoma, non-cancerous tissues often exhibit detectable DNA methylation changes that are more evident than genetic alterations. Therefore, alterations in DNA methylation may play a more critical role in the field cancerization of the urothelium 40. A meta-analysis showed that in BC tissues and adjacent normal tissues, the promoter of the death-associated protein kinase (DAPK) gene, located on chromosome 9p34 and involved in the p53-dependent apoptosis pathway, was methylated at similar frequencies, and this was significantly associated with the occurrence of BC 41. Similarly, in bladder tissues from patients with neurogenic lower urinary tract dysfunction, varying degrees of DNA methylation have been observed in the promoters of tumor-related genes, including Ras association domain family 1, retinoic acid receptors beta, DAPK, human telomerase reverse transcriptase, and adenomatous polyposis coli. This methylation is closely associated with the increased risk of recurrent urinary tract infections and further supports the presence of the epigenetic field effect, suggesting potential risk for BC development 42. These hypermethylated genes typically lead to the inactivation of tumor suppressor genes, allowing tumor cells to escape normal cellular regulation, and thereby promoting cancer development 43. However, it is noteworthy that some genes, such as distal-less homeobox 6 and specificity protein 8, exhibit hypomethylation in the field effect. These genes are often related to embryonic development, and tumor cells may activate developmental pathways to enhance their survival and proliferation 16. In NMIBC, the presence of hypomethylation, unlike in invasive tumors, may provide a biological explanation for its non-invasive behavior 44.

Tadeusz Majewski et al. proposed that widespread mucosal methylation changes may represent an early field alteration for bladder cancer. It suppresses immune responses, and disrupts Ras signaling pathways, leading to the enhanced growth and spread of mutated cells within normal mucosa. Furthermore, signaling pathways affected by DNA copy changes and mutations, such as the Kras pathway, precede the onset of DNA methylation, suggesting that they may enhance mechanisms initiated by methylation 23. Sangkyou Lee et al. developed a method combining histology and molecular mapping to resolve the initial clonal expansion sequence prior to tumorigenesis. They find that, prior to the loss of tumor suppressor gene function, five genes associated with the field effect of BC located within a 5 Mb region surrounding the retinoblastoma susceptibility gene were downregulated. Further analysis of the lysophosphatidic acid receptor 6 and calcium-binding protein 39-like involved in the luminal and basal subtypes of BC respectively, shows that these genes are silenced by hypermethylation of the CpG site in the promoter, preventing transcription factor binding, affecting cholesterol metabolism and the unfolded protein response, leading to dysregulation of uroepithelial differentiation and promoting cancer progression 45. However, whether methylation is a true initiating event remains unresolved because most available human data are cross-sectional and observational. Thus, current evidence should be interpreted as suggesting early epigenetic involvement and highlighting the need for longitudinal sampling and functional perturbation studies.

Reported methylation alterations in field cancerization are assay dependent, because targeted MSP/qMSP prioritize predefined loci and maximize analytical sensitivity in low-input urine DNA, whereas Illumina HM450/EPIC arrays and bisulfite-based sequencing (e.g., WGBS) profile broader CpG sets with different coverage and technical biases. As a result, cross-study comparisons and consensus marker selection can be confounded by platform- and workflow-specific effects 46.

Beyond DNA methylation, histone modification and chromatin organization may also contribute to field cancerization. KMT2C and KMT2D are of particular interest because they are frequently inactivated in urothelial carcinoma and, in some studies, in histologically normal-appearing urothelium. These genes encode components of the H3K4me1 methyltransferase machinery.

When KMT2C or KMT2D function is lost, enhancer regulation can be disrupted. This may weaken transcriptional programs involved in urothelial differentiation and is associated with reduced luminal features and expansion of basal-like cell populations. Altered KMT2C/KMT2D activity may also affect the distribution of the KMT2A-menin complex. Rather than remaining at shared enhancers, this complex can become enriched at promoters with bivalent chromatin marks, including H3K4me3 and H3K27me3.

Such redistribution may relieve repression of genes that are normally tightly controlled, particularly immediate early genes and genes involved in inflammatory signaling. In this setting, urothelial cells may become less differentiated and more responsive to growth-related or inflammatory cues. These findings suggest that chromatin remodeling may not directly initiate tumor formation by itself, but could leave the urothelium in a more permissive state for later genetic alterations or continued carcinogen exposure 47.

### 3.3 Dysregulation of transcription, protein expression, and metabolism

Transcriptomic studies have identified clear molecular differences between urothelial carcinoma subtypes, and these differences may also be relevant to the evolution of field changes 16, 48. Basal-type bladder cancers are more often associated with invasive disease and commonly express keratin 5 and keratin 6. These tumors also show stronger activation of epithelial-mesenchymal transition (EMT)-related programs. TGF-β1 can promote EMT, whereas p53 has been reported to restrain this process. Transcription factors from the SNAIL, TWIST, ZEB, and FOX families participate in this shift by reducing epithelial markers such as E-cadherin, claudin-1, and tight junction protein 1. TP63 is also involved in the regulation of basal keratins and EMT-associated transcriptional programs in urothelial cells. By comparison, luminal tumors, which are more commonly linked to NMIBC, generally show weaker EMT activity and higher expression of GATA3 16. These transcriptional patterns are relevant clinically. Basal programs are usually associated with greater invasiveness and more aggressive biological behavior, whereas luminal programs are more closely related to urothelial differentiation and may show different treatment responses 49.

Proteomic and multi-omics studies further indicate that metabolic regulation, immune activity, and protein homeostasis differ across UC subtypes and disease stages. In one analysis comparing papillary urothelial carcinoma (PUC) with carcinoma in situ (CIS), PUC showed increased FOXO1 and AKT activity, with downstream effects on genes involved in glucose metabolism. CIS, in contrast, showed marked upregulation of JUN and enrichment of pathways related to EMT and immune regulation. Increased infiltration of CD8⁺ T cells, dendritic cells, and M1 macrophages was also observed in CIS.

Metabolic features differed between the two trajectories. In PUC, increased glycolysis and lactate production were negatively associated with immune scores, raising the possibility that lactate-rich conditions may contribute to local immune suppression. During CIS progression, pathways involved in reactive oxygen species metabolism were reduced, while DNA damage-response pathways were increased. This combination may reflect persistent oxidative and genotoxic stress, which could favor genomic instability during progression 50. The proteogenomic distinction between PUC-derived and CIS-derived trajectories has potential clinical relevance because it maps molecular programs onto progression risk. Another study reported that NMIBC tissues upregulate stress-adaptive and metabolic support proteins such as peroxiredoxin 1, glutathione S-transferase mu 1, and heat shock protein beta-1, whereas MIBC shows prominent increases in a different set of redox homeostasis regulators, including peroxiredoxin 2, biliverdin reductase B, and 15-hydroxyprostaglandin dehydrogenase. This pattern points to stage-dependent changes in antioxidant responses rather than a single, uniform redox program across UC progression 51 Czerniak and colleagues examined protein expression and metabolic alterations in mucosa adjacent to bladder dysplasia and carcinoma in situ 52. Their findings suggest that molecular disruption is already present before invasive carcinoma becomes apparent. Several pathways involved in energy production, including oxidative phosphorylation, branched-chain amino acid degradation, the tricarboxylic acid cycle, and thermogenesis, were reduced. Together, these changes are consistent with impaired mitochondrial and metabolic capacity.

The study also identified abnormalities in nucleocytoplasmic transport, spliceosome function, ribosome biogenesis, and peroxisomal activity. These findings indicate that protein synthesis, processing, and turnover may already be disturbed in the altered mucosa. Changes in these pathways were linked to dysregulation of peroxisome proliferator-activated receptor signaling, which is relevant to urothelial differentiation, lipid handling, and protein degradation.

Metabolomic data further showed alterations in cysteine and methionine metabolism, fatty acid metabolism, and glycolytic activity. Rather than representing isolated metabolic events, these changes may reflect a broader disturbance in glucose and lipid utilization within the local mucosal environment. Such an altered metabolic background could contribute to the field effect and make the urothelium more permissive to subsequent malignant transformation.

Notably, whole-organ spatial proteomic and metabolomic mapping identified increased lactate levels and enhanced glycolytic activity in microscopically normal-appearing mucosa adjacent to dysplasia or carcinoma in situ. These changes were detectable before overt carcinoma developed. This observation suggests that a Warburg-like metabolic shift may emerge at an early stage of urothelial field cancerization and provides a potentially testable link between metabolic remodeling and later tumor development.

### 3.4 Microenvironmental Changes

During tumorigenesis and progression, although molecular genetic alterations are extensively studied, the role of the tumor microenvironment is equally critical. The tumor microenvironment consists of various components, including non-cellular elements such as extracellular matrix, bioactive factors, and extracellular vesicles, as well as stromal cells such as fibroblasts, adipocytes, endothelial cells, and immune cells. These factors collectively influence tumor cell survival, invasion, and metastasis 53-55. In the absence of microenvironmental signals such as inflammation, clonal expansion is unlikely to occur even when mutations are present 56.

The immune-inflammatory response plays a significant role in field carcinogenesis. Jolanta Bondaruk et al. found that basal BC exhibits several mutated genes associated with the inflammatory response, such as the toll-like receptor 4/5, which recognize pathogens and activate innate immune responses, as well as phosphoinositide-3-kinase adapter protein 1, which is involved in the survival of mature B cells and negatively regulates the production of inflammatory cytokines. At the same time, widespread dysregulation of interleukin-related pathways persists during the progression from normal urothelium to urothelial carcinoma 16. Moreover, in the ostensibly normal mucosal stroma of bladder cancer patients, the numbers of stellate cells and mast cells are reduced. These stromal cells contribute to local immune regulation. Their reduced abundance in apparently normal mucosa may weaken antitumor surveillance and favor a more immunosuppressive environment 57. The field effect also involves adaptive immune pathways. Basal bladder cancer exhibits mutations in zeta-chain associated protein kinase 70 and POU domain class 2-associating factor 1, which are major regulators of T- and B-cell development, revealing microenvironmental effects on immune cell function and development 16. Strandgaard et al. assessed CD8⁺ T-cell exhaustion using a transcriptomic score based on the expression of immunoinhibitory genes in tumor RNA-seq data. CD8⁺ T-cell infiltration was estimated computationally. Their results suggested that persistent antigen exposure may contribute to progressive T-cell exhaustion during field carcinogenesis, which could reduce antitumor immune activity and favor tumor progression 15 Overall, tissue-based studies point to an altered local immune environment that may be more permissive for tumor development. Experimental studies of extracellular vesicles (EVs) provide possible mechanisms through which tumor cells may influence adjacent urothelium.

EVs released by bladder cancer cells have been reported to induce lasting changes in susceptible urothelial cells and, under experimental conditions, may promote malignant transformation 58. Electronic-cigarette exposure can increase EV release from bladder cancer cells. These EVs have been associated with oxidative stress, inflammatory signaling, DNA damage, and increased proliferation in non-malignant urothelial cells 59 Several molecular pathways may be involved. EV-mediated activation of the ERK pathway has been linked to increased expression of CREPT, a transcription-associated protein that can promote chromatin looping and cell-cycle-related transcription. Transcriptomic analyses also suggest that EV exposure activates TNF-related inflammatory signaling. CREPT may further influence this response by regulating TNFR2 and PI3K expression, potentially connecting chronic inflammation with pro-tumorigenic changes in recipient cells 60 The protein cargo carried by tumor-derived EVs may also contribute to these effects. PDIA1, an endoplasmic reticulum oxidoreductase and chaperone protein, has been identified as a potentially important EV component in bladder cancer models. Under endoplasmic reticulum stress, tumor cells may selectively release oxidized and active PDIA1 through EVs. Although this export may reduce intracellular oxidative stress in the donor tumor cells, PDIA1-containing EVs can have different effects after uptake by normal urothelial cells. Experimental evidence indicates that they may induce sustained oxidative stress and DNA damage in recipient cells. Repeated exposure has been associated with malignant transformation and anchorage-independent growth *in vitro* 61. Morphologically, exposure to tumor-derived EVs induces significant changes in urothelial cells, including cytoplasmic enlargement, disrupted endoplasmic reticulum (ER) organization, and mitochondrial accumulation. These changes trigger the unfolded protein response in the ER (UPRER). Prolonged UPRER signaling activates survival branches, leading to significant upregulation of inositol-requiring enzyme 1, nuclear factor-kappaB, and the inflammatory cytokines, ultimately suppressing the expression of the pro-apoptotic protein C/EBP homologous protein. These alterations increase genomic instability, disrupt contact inhibition, and enhance cellular invasiveness, creating conditions favorable for tumor progression and dissemination 58. Taken together, EV-driven oxidative/ER-stress phenotypes provide testable mechanisms for field carcinogenesis, but the strength of evidence currently differs markedly between mechanistic model systems and clinical association studies.

EV-associated signals are attractive for translation in bladder cancer because urine is readily accessible and repeatedly sampled. However, urinary EVs are heterogeneous and can originate from the kidney or upper tract and non-malignant urothelium, potentially diluting bladder-tumor-derived signals. Thus, study design should consider specimen type when “functional” EV biomarkers are pursued 62. Consistent with this, recent studies and reviews summarize candidate urinary EV cargo classes—including miRNAs, proteins, and glycan/glycoprotein features—for diagnosis, classification, or monitoring of bladder cancer 63. Yet, reproducibility is limited by variability in EV isolation, characterization and pre-analytical factors. Adherence to community reporting standards and transparent EV characterization will be essential 64.

## 4. Implications of Field Cancerization for Clinical Strategies

In recent years, driven by the rapid advances in high-throughput sequencing and related technologies, researchers have progressed from bulk sequencing at the population level to single-cell omics analyses that reveal cellular heterogeneity, and further to spatial omics that preserve positional information. This progression enables comprehensive profiling of cell populations within FC regions across the genomic, epigenomic, transcriptomic, proteomic, and metabolomic levels. It can help clarify how molecular changes contribute to UC initiation, progression, and interactions with the surrounding microenvironment, with possible implications for earlier detection and more individualized treatment 65, 66. Multi-timepoint spatial and temporal omics approaches may further extend this work. Unlike single-timepoint maps, these methods may help track changes in cell populations and local signaling over the course of field cancerization. They could also provide insight into cell-state transitions and evolving interaction networks within altered urothelium and its microenvironment 67, 68. The major molecular alterations reported in UC-related field cancerization are summarized in Table 1.

Field effects are generally viewed as a source of risk because altered urothelial areas may provide a background for multifocal tumor formation. However, mutated clones in normal tissue do not necessarily behave in the same way as tumor clones. In some settings, clonal competition within normal epithelium may remove or suppress early neoplastic cells 69 The expansion of certain mutant populations may therefore reflect a tissue-level selection process rather than inevitable malignant progression. Although this process can increase the number of detectable mutations in apparently healthy tissue, it may occasionally limit the growth of more harmful clones 70. This complexity should be considered when interpreting molecular abnormalities in cancer-prone fields.

From a translational perspective, field cancerization may offer opportunities for risk assessment, early detection, and follow-up after treatment. Smoking, chronic inflammation or infection, dietary factors, chemical exposure, and physical carcinogenic stimuli are all relevant to the development of altered urothelial fields, which also emphasizes the value of prevention 71, 72. Clinically, applying optical imaging technologies to develop fluorescent probes that specifically bind early gene mutations or DNA methylation sites could enable precise localization of FC areas and early tumor detection under cystoscopy. Clinical studies have supported that hexaminolevulinate-guided fluorescence cystoscopy can improve detection and reduce early recurrence compared with white light cystoscopy alone, indicating that optical enhancement can be clinically actionable when integrated into standard cystoscopic workflows 73. Building beyond this precedent, near-term optical probe development is generally more feasible when targeting accessible cell-surface proteins, whereas urine-based mutation and methylation assays can serve as noninvasive tools for risk enrichment to define high-risk patients or fields 74, 75. In this context, candidate targets with translational evidence include CD47-directed fluorescence imaging in human bladder tissues and a CD44v6-targeted near infrared fluorescent agent evaluated in a first-in-human endoscopic study 76, 77. In addition, clinical translation of targeted optical probes will require prospective evaluation of safety and diagnostic accuracy within standardized imaging protocols and an appropriate regulatory framework for agent-based imaging during cystoscopy. Targeting microenvironmental changes may facilitate novel immunotherapeutic agents to reduce tumor recurrence 5, 78-80. However, due to the limited availability of clinical specimens, incorporating FC research into animal models is essential. By performing multi-site, longitudinal sampling to dynamically analyze molecular evolution from normal through precancerous to tumor stages, we can optimize model systems that recapitulate human lesion progression, facilitating the development of urothelial carcinoma animal models that closely resemble human cancer progression and support clinical drug research 70.

Recent studies indicate that urinary tumor DNA (utDNA) and circulating tumor DNA (ctDNA) hold promise as prospective tools for detecting minimal residual disease and metastatic recurrence. Both approaches may detect molecular residual disease more sensitively than conventional pathological assessment. Detectable utDNA or ctDNA may originate from overt tumor cells, occult microscopic disease, or molecularly altered cells within histologically normal-appearing urothelium. Higher utDNA and ctDNA levels have also been associated with unfavorable clinical outcomes and an increased risk of recurrence 15, 81-83 These assays may provide additional information during surveillance and could eventually reduce reliance on frequent cystoscopy in selected patients. Their limitations should nevertheless be recognized. A negative liquid-biopsy result can be difficult to interpret when tumor-derived DNA is present at very low levels, as assay sensitivity declines with low analyte abundance 84. Plasma ctDNA testing may also be affected by clonal hematopoiesis, which can introduce biological noise and false-positive signals. Matched leukocyte sequencing or appropriate filtering methods may help address this issue 85 In a cohort of patients undergoing radical cystectomy with curative intent, utDNA-based minimal residual disease detection predicted pathological response with 81% sensitivity and 81% specificity 86. These results support the clinical potential of utDNA testing, although a negative result should not be considered definitive evidence that residual disease is absent.

## 5. Current challenges and future prospects

Field cancerization has broadened the understanding of urothelial carcinoma development, but several practical and conceptual issues remain unresolved. Spatial and temporal transcriptomic methods can map cell populations and transcriptional programs within tissue, yet their broader use is still limited by cost, technical demands, and the scale of data processing required. Many available studies are based on cross-sectional samples and use computational trajectory analysis to infer cell-state transitions 67. Such pseudotime analyses are useful, but they cannot replace prospective longitudinal sampling. Large datasets that include repeated sampling and precise anatomical information are still scarce 87. Future studies will need designs that balance molecular depth with feasible cohort sizes. When possible, whole-organ mapping or geographically documented sampling should be incorporated to clarify how far field-associated alterations extend within the urothelium 23.

Tissue collection presents another challenge. Repeated biopsies from several sites may increase patient burden and raise ethical concerns, particularly when samples are taken from areas that appear normal on routine pathology. Small differences in sampling location can also affect molecular findings and make comparisons between studies difficult 88, 89. One practical solution may be the use of standardized biobanking procedures. Preserving residual mucosa from surgical specimens, together with detailed information on sampling location, could reduce the need for additional research biopsies and improve reproducibility across cohorts. It is also important to note that most current evidence comes from bladder cancer. Direct evidence for field cancerization in upper tract urothelial carcinoma remains limited, and findings from bladder-based studies should not be automatically applied to upper tract disease without further validation.

Experimental models also have limitations. Cell culture systems and conventional animal models cannot fully reproduce the complex epithelial, stromal, immune, and spatial interactions present in the human urothelial field 90-93. Animal studies should therefore be interpreted as models of early mucosal alteration rather than exact representations of human field cancerization. For example, introducing Trp53 mutations into Krt5-expressing basal cells increased the incidence of muscle-invasive bladder cancer in a BBN-induced model, indicating that lineage-specific genetic changes can interact with chronic carcinogen exposure to influence disease progression 94.

A further issue is the lack of a widely accepted definition of field cancerization in urothelial carcinoma. The concept was initially developed in head and neck cancer research, and its application to urothelial tumors remains inconsistent. Studies often focus on molecular abnormalities in tissue adjacent to tumors but do not always address the wider anatomical distribution or clinical relevance of these changes 95, 96. Future work would benefit from reporting sampling sites in a standardized and geographically explicit manner. Important analytical parameters should also be predefined and reported clearly. Where appropriate, data should be deposited in controlled-access repositories so that findings can be independently examined while protecting patient privacy.

Progress in this area will likely depend on three related developments. First, a practical definition of field cancerization is needed that combines histological appearance with anatomical location and molecular features. Second, prospective studies with geographically documented sampling are required to improve comparability between cohorts. Third, translational approaches should be tested in clinical settings, particularly urine-based molecular assays, imaging methods, and prevention-oriented interventions. The priority should not simply be to generate more descriptive data, but to determine whether field-associated molecular changes can improve patient stratification, surveillance, or treatment decisions.

## 6. Conclusions

Current evidence indicates that field cancerization is a relevant biological feature of urothelial carcinogenesis rather than only a descriptive observation. Genetic, epigenetic, metabolic, and microenvironmental abnormalities can be detected in urothelium that appears normal by conventional histology, suggesting that molecular changes may arise before an overt lesion is visible.

Field cancerization does not replace the clonal expansion model. Instead, it offers an additional explanation for multifocal disease, recurrence, and molecular heterogeneity between urothelial lesions. Its potential clinical value lies mainly in molecular risk assessment and in the development of surveillance tools based on urine testing or imaging. However, routine application will require standardized definitions, consistent sampling strategies, and prospective validation in clinically representative cohorts.

Overall, field cancerization provides a useful framework for understanding how altered urothelial tissue may contribute to tumor development and recurrence. Further work should focus on translating these observations into approaches that support earlier detection, more accurate risk assessment, and better prevention of recurrence.

## Figures and Tables

**Table 1:** Molecular alterations associated with field cancerization in urothelial carcinoma

Molecular Alteration	Evidence type	Sample	Summary	Ref
Gene alterations, microenvironmental changes, urinary proteome changes	Human multi-site tissue sequencing and urine biomarker study	High-risk NMIBC treated with BCG: 751 selected-site bladder biopsies (662 normal-appearing urothelium, 79 lesions) from 70 patients; 234 urine samples from 112 patients; 223 tumors from 136 patients.	Error-corrected sequencing identified mutations in normal-appearing urothelium, including KDM6A, ARID1A, and TP53. High field level linked to CD8 T-cell exhaustion and worse post-BCG outcomes; utDNA after BCG predicted recurrence/progression and high field increased VEGFA and CD27.	15
Gene alterations	Human longitudinal tumor evolution study	Serial TURBT specimens from 73 patients progressing to MIBC, metastasis, or radical cystectomy: 357 FFPE tumors; 271 tumors for immunohistochemistry subtyping; 200 tumors for gene-expression profiling; 220 tumors for hotspot genotyping.	Longitudinal analysis showed that hotspot FGFR3, PIK3CA, and TERT promoter alterations were largely preserved across recurrences, whereas progression toward more aggressive subtypes was associated with abnormal p53 staining.	29
Gene alterations	Human normal-appearing urothelium sequencing/mapping study	Laser-microdissected histologically normal urothelium from 15 transplant organ donors (1,647 microbiopsies) and 5 bladder cancer patients (450 microbiopsies); targeted 321-gene panel, WES, and WGS.	Patchwork of microscopic clones (median 40 exome/1879 genome mutations); 17 positively selected genes dominated by chromatin remodeling; APOBEC-positive microbiopsies 22%; TP53 rare, FGFR3 and TERT promoter absent.	27
Gene alterations	Human tumor genomic association study	82 bladder tumors analyzed by targeted NGS: 41 with prior pelvic radiotherapy for prostate cancer and 41 without prior radiotherapy.	Recurrent variants showed CDKN2A mutation enriched in non-radiotherapy controls, while ATM enriched in 0-10y post-radiotherapy and KDM6A enriched ≥10y.	34
Gene alterations	Human normal-appearing margin chromosomal analysis study	Primary tumors, matched histologically normal urothelial margins sampled 1-2 cm (proximal) and 4-5 cm (distal) from the tumor, and whole blood from 67 patients (145 tumors, 277 margins, and 63 blood samples).	SNP-array/MoChA detected low-fraction post-zygotic chromosomal gains, deletions, and copy-neutral loss of heterozygosity in 45% of patients' histologically normal margins; 16 recurrent hotspots overlapped bladder cancer driver regions, supporting field cancerization beyond histology.	22
Gene alterations	Human normal-appearing urothelium sequencing study	Laser-capture microdissected morphologically normal bladder/ureter urothelium (161 samples from 120 patients), with matched tumors (n = 126) and blood; whole-exome sequencing.	Morphologically normal urothelium contained macroscopic mutant clones, with frequent KMT2D and KDM6A alterations but rare TP53, PIK3CA, and FGFR3 mutations.	26
Gene alterations	Human targeted sequencing study of normal urothelium and paired NMIBC	TURBT tissues from 90 BCG-naive NMIBC patients (124 tissues) and 34 post-BCG relapse patients; deep targeted sequencing with matched normal.	TERT promoter and chromatin-remodeling alterations were relatively stable, whereas FGFR3, PIK3CA, TSC1, and TP53 changed more dynamically over time. ARID1A mutation was associated with relapse risk, and CCNE1 amplification with progression risk, supporting persistence of altered precursor populations with ongoing evolution after BCG.	28
Epigenetic alterations	Human methylation study of non-cancerous urothelium	Non-cancerous urothelium from UC patients (N = 47) with paired tumors (T = 46) and control urothelium (C = 26); genome-wide DNA methylation profiling.	2,502 differentially methylated CpG probes between non-cancerous urothelium and controls. Many of these alterations were retained or strengthened in paired tumors.	40
Epigenetic alterations	Human methylation study in non-malignant bladder mucosa	Cold-cup bladder biopsies from patients with neurogenic lower urinary tract dysfunction (n = 24); qMSP analysis.	RARB, RASSF1, and DAPK promoter hypermethylation was frequent.	42
Gene, epigenetic, and transcriptional alterations	Human whole-organ spatial multi-omics mapping study	Normal urothelium, low-/high-grade intraurothelial neoplasia, and UC from 9 cystectomy bladders; Luminal map24 and basal map19 with ureter normal controls and matched blood germline.	Spatial multi-omics identified early field changes across anatomically mapped mucosa, including aberrant CpG methylation, monotonic expression plaques, and expanding clonal mutations/CNVs.	16
Epigenetic alterations	Human methylation study of matched normal-appearing urothelium	49 Ta-T1 and 38 T2-T4 UC; matched normal-appearing urothelium microdissected (≥5 cm from invasive tumor); 12 cancer-free urothelium controls.	Ta-T1 tumors were hypomethylated, whereas T2-T4 tumors were broadly hypermethylated; matched normal-appearing urothelium already showed hypermethylation at about 12% of loci, supporting a polyclonal epigenetic field defect consistent with FC.	44
Gene and epigenetic alterations	Human whole-organ methylation field-mapping study	Single cystectomy bladder mapped into 41 1×2 cm blocks; scraped mucosa or microdissected UC (>90% urothelial/tumor cells); matched blood DNA; methylation controls: ureter urothelium from 3 nephrectomies.	Field-effect mucosa (normal urothelium/low-grade intraurothelial neoplasia) showed early driver mutations (TERT, KDM6A, ARID1A) and widespread CpG-island dysmethylation, with low-level CNVs.	23
Epigenetic alterations	Human whole-organ promoter methylation/LOP mapping study	Whole-organ cystectomy maps (n=20): bladder mucosa partitioned into 1×2 cm blocks (902 geo-annotated samples; plus primary tumors (n=189) and normal urothelium controls; promoter methylation by quantitative methylation-specific PCR/bisulfite sequencing; LOP around RB1 by SNPlex/SNP allelotyping.	Plaque-like promoter hypermethylation silenced forerunner genes LPAR6 or CAB39L and extended into NU with minimal/no morphologic change, inversely correlating with expression. LPAR6 and CAB39L were methylated in 72% and 92% of tumors, biasing urothelial basal-to-luminal differentiation toward luminal/papillary vs basal states.	45
Gene and transcriptional alterations	Human longitudinal tumor evolution study	Serial primary and recurrent bladder tumors (18 specimens) from 5 patients, including locoregional and distant metastases, with matched blood; whole-exome sequencing and RNA sequencing.	Recurrent lesions largely retained early clonal driver mutations but acquired additional subclonal expansions, and RNA sequencing indicated switching between basal and luminal expression subtypes over time and sites, consistent with evolving residual populations driving relapse.	48
Transcriptional and metabolic alterations	Human multi-omics proteogenomic study	FFPE urothelial bladder lesions across stages from 190 UC patients; proteome n=448, phosphoproteome n=211, whole-exome n=125, transcriptome n=67.	Papilloma was dominated by HRAS hotspot mutations (83%) with MAPK activation and mTORC1 pathway dysregulation. PUC showed higher glucolipid metabolism and lower immune infiltration, whereas CIS showed DNA damage response, APOBEC signature, RBPMS loss, and activator protein 1 (AP-1) activation.	50
Proteomic alterations	Human tissue proteomic comparison study	UBC tumor tissue (NMIBC Ta/T1 n=25; MIBC T2-T4 n=17) and noncancerous bladder tissue (n=10); proteomic profiling.	Twelve proteins differed, 9 higher in NMIBC and 3 higher in MIBC, dominated by oxidative stress and antioxidant functions, including PRDX1/PRDX6 versus PRDX2, biliverdin reductase B, and 15-hydroxyprostaglandin dehydrogenase, suggesting redox adaptation as candidate biomarkers.	51
Proteomic and metabolic alterations	Human whole-organ spatial proteomic/metabolomic mapping study	Radical cystectomy, whole-organ grid sampling across mucosa and tumor; DNA, proteome, and metabolome from each well; whole-exome sequencing plus LC-MS/MS proteomics and targeted LC-MS/MS metabolomics.	Across normal urothelium, low- and high-grade intraurothelial neoplasia, and invasive UC, protein homeostasis pathways and PPAR-regulated lipid/carbohydrate metabolism were dysregulated; mutations clustered by spread and VAF into alpha, beta (below 20%), and gamma (above 20%) patterns, with gamma expanding.	52
Microenvironmental alterations	Human tissue immunohistochemistry study	Bladder neoplasia (n=52) and benign bladder conditions (n=18); benign-appearing mucosa and neoplastic tissue; immunohistochemistry for ALDH1A1 and CD44 plus differentiation markers.	Compared with benign controls, benign-appearing mucosa from UC patients showed fewer ALDH1A1-positive stromal stellate cells and mast cells, consistent with stromal field-effect remodeling near cancer.	57
Microenvironmental alterations via extracellular vesicles	Cell/extracellular vesicle transformation model	SV-HUC urothelial cells treated twice weekly with bladder cancer cell-derived EVs (20 μg/mL, 8-13 weeks; 12 weeks plus 4-week washout), including cigarette-smoke extract (1%) or unflavored/menthol E-liquid-stimulated EVs from TCCSUP/T24; soft agar colonies and athymic nude-mouse xenografts.	EV uptake induced endoplasmic reticulum stress with sustained unfolded protein response signaling (PERK to IRE1 shift, NF-kappaB activation), inflammatory cytokine release, oxidative stress, and DNA damage, leading to contact-inhibition escape, invasion, and anchorage-independent growth, consistent with FC-driven transformation.	58, 59
